# The selective GSK3 inhibitor, SAR502250, displays neuroprotective activity and attenuates behavioral impairments in models of neuropsychiatric symptoms of Alzheimer’s disease in rodents

**DOI:** 10.1038/s41598-019-54557-5

**Published:** 2019-12-02

**Authors:** Guy Griebel, Jeanne Stemmelin, Mati Lopez-Grancha, Denis Boulay, Gerald Boquet, Franck Slowinski, Philippe Pichat, Sandra Beeské, Shinji Tanaka, Akiko Mori, Masatake Fujimura, Junichi Eguchi

**Affiliations:** 1 Sanofi, Strategy & Business Development, Chilly-Mazarin, France; 2 Sanofi R&D, Global Project Management, Chilly-Mazarin, France; 3 Sanofi R&D, Rare and Neurologic Disease Research, Chilly-Mazarin, France; 4 Sanofi R&D, Translational In Vivo Models, Chilly-Mazarin, France; 5 Sanofi R&D, Integrated Planning and Operations, Project Planning Management, Chilly-Mazarin, France; 6 Sanofi R&D, Integrated Drug Discovery, Chilly-Mazarin, France; 7 Sanofi R&D, Integrated Planning and Operations Management, Chilly-Mazarin, France; 8 Sanofi R&D, Cardiovascular Diseases & Metabolism, Chilly-Mazarin, France; 9 Research Division, Mitsubishi Tanabe Pharma Corporation, Kamoshida-cho, Aoba-ku, Yokohama Japan

**Keywords:** Alzheimer's disease, Pharmacology

## Abstract

Glycogen synthase kinase 3 (GSK3) has been identified as a promising target for the treatment of Alzheimer’s disease (AD), where abnormal activation of this enzyme has been associated with hyperphosphorylation of tau proteins. This study describes the effects of the selective GSK3 inhibitor, SAR502250, in models of neuroprotection and neuropsychiatric symptoms (NPS) associated with AD. In P301L human tau transgenic mice, SAR502250 attenuated tau hyperphosphorylation in the cortex and spinal cord. SAR502250 prevented the increase in neuronal cell death in rat embryonic hippocampal neurons following application of the neurotoxic peptide, Aβ_25–35_. In behavioral studies, SAR502250 improved the cognitive deficit in aged transgenic APP(SW)/Tau(VLW) mice or in adult mice after infusion of Aβ_25–35_. It attenuated aggression in the mouse defense test battery and improved depressive-like state of mice in the chronic mild stress procedure after 4 weeks of treatment. Moreover, SAR502250 decreased hyperactivity produced by psychostimulants. In contrast, the drug failed to modify anxiety-related behaviors or sensorimotor gating deficit. This profile confirms the neuroprotective effects of GSK3 inhibitors and suggests an additional potential in the treatment of some NPS associated with AD.

## Introduction

Neuropsychiatric symptoms (NPS) are frequently observed in patients with Alzheimer’s disease (AD) and are being increasingly recognized as hallmarks of this condition and related dementias^1^. These symptoms range from aggression, anxiety, cognitive deficit, depression, disinhibition, irritability, sensorimotor deficit and sleep disorders with a global prevalence of 39 to 49 percent for the most frequent NPS^2^. The presence of NPS in the early stages of AD predicts disease progression^3^. In addition, there is only one approved drug for the treatment of NPS in AD in Europe and Canada, namely risperidone for the management of aggression. A range of medications, including monoaminergic antidepressants, benzodiazepine anxiolytics, cholinesterase inhibitors or the psychostimulant methylphenidate, have been occasionally used for the treatment of NPS in AD patients but they have demonstrated limited efficacy and sometimes poor compliance due to unwanted adverse effects (for a recent review, see^1^). As a result, there is renewed interest in finding more effective treatments for NPS in AD^1^.

A drug combining disease-modifying activity with NPS-reducing potential would represent an ideal therapy for the treatment of AD. Among the most interesting candidates bearing the potential of targeting the disease at its roots along with its associated NPS are glycogen synthase kinase-3 (GSK3) inhibitors. GSK3 which was discovered about 30 years ago is a serine/threonine protein kinase involved in a variety of cellular processes, e.g., microtubule dynamics, gene transcription and cell proliferation^4–6^. GSK3 is involved in AD progression, including pathophysiological formation of paired helical filament tau, an integral part of the deposits of neurofibrillary tangle responsible for disruption of neuronal function in this condition^7^. Hence, inhibiting this protein kinase has been suggested to be a potential strategy to treat patients suffering from AD (see^8,9^ for two recent reviews). In preclinical models, lithium an early GSK3 inhibitor was found to reduce aspects of AD pathology, including tau phosphorylation and amyloid production both *in vitro* and *in vivo*^7,10–12^. During the last decade, several selective, orally active and brain penetrant GSK3 inhibitors have been identified. They have demonstrated neuroprotective activity by inhibiting hyperphosphorylation of tau protein in cell-based assays (for a recent review, see^9^).

Evidence supporting the idea that GSK3 inhibitors may have an additional potential for alleviating NPS originates from several studies in animals showing that GSK3 inhibition may contribute to the action of antidepressants^13–17^ and antipsychotics^18–23^. This idea is additionally substantiated by studies with selective GSK3 inhibitors, which showed that these drugs produce antidepressant-like effects in animal models^24–27^. Evidence supporting a therapeutic potential of these molecules in other NPS, such as anxiety or agitation has not been well established.

**Figure 1:**
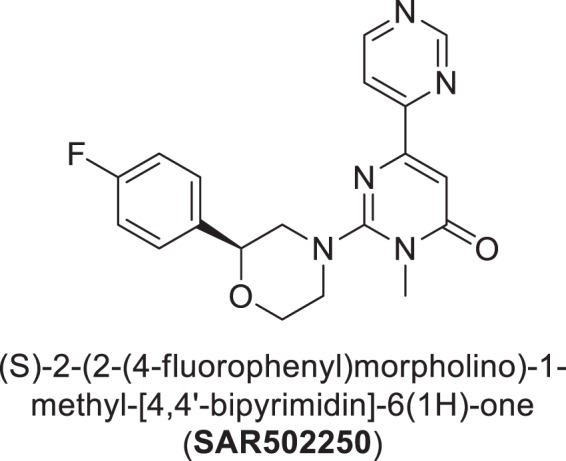
Chemical structure of SAR502250.

In this context, the objective of this study was to characterize the behavioral effects of the selective ATP competitive GSK3 inhibitor, SAR502250 (a.k.a. UDA-680) (Fig. 1), in models related to certain aspects of NPS, including agitation, aggression, anxiety, cognitive and sensorimotor deficits, and depression. The compound was additionally tested for its neuroprotective potential in *in vitro/vivo* assays of cell death and tau hyperphosphorylation. SAR502250 was described previously as a potent, selective and competitive inhibitor of mouse and human GSK3 (IC_50_ = 12 nM in both species), with excellent brain permeability in the mouse (brain/plasma ratio: 2.7 after 2 hours)^28,29^.

## Methods and Materials

### Ethics statement

All experimental procedures described herein were carried out in accordance with the “Guide and Care” and were approved by the Animal Ethics Committee of Sanofi and Institutional Animal Care and Use Committee of Research Laboratories, Mitsubishi Tanabe Pharma Corporation.

### Animals

Animals had access to food and water *ad libitum* with a 12-h light/dark cycle (lights on at 7:00 a.m.). The following species and strains were used: (1) mice: BALB/c, C57BL/6J, CD1, OF1 and Swiss (Charles River Laboratories, Janvier Labs, Le Genest Saint Isle, France or Iffa Credo, Les Oncins, France), APP (SW)/Tau (VLW) and P301L human tau transgenic mice (Taconic Biosciences); (2) Rats: Wistar and Sprague-Dawley (Iffa Credo) (see below for further details). Different species and strains were used on the basis of pilot experiments, which demonstrated that some species and/or strains are more suitable than others in certain models. Tests were performed during the light (day) cycle.

### Drugs

SAR502250 (Sanofi Medicinal Chemistry), amphetamine, fluoxetine, lithium chloride, phencyclidine (PCP) (Sigma-Aldrich, Saint-Quentin Fallavier, France) were dissolved or suspended in distilled water with 0.6% methylcellulose and the addition of 5% Tween 80 (Sigma-Aldrich) or 2% Cremophor in *in vivo* studies and suspended in dimethylsulfoxyde (DMSO) at 10 mM in *in vitro* experiments. Doses refer to the weight of the free base. SAR502250 was administered orally (*per os*, p.o.) in the behavioral tests with the exception of the chronic mild stress procedure where it was administered intraperitoneally (i.p.). This administration route was chosen because exploratory experiments showed that it is more suitable in this test than the p.o. route. Different treatment schedules were chosen because some of the procedures used required repeated administration to observe a drug effect (e.g. chronic mild stress). The amount of vehicle was adjusted to be the same for all the doses and controls. Volume of administration was 10 or 20 ml/kg in mice, 1 or 5 ml/kg in rats. All drug solutions were prepared fresh daily.

### Characterization of SAR502250 in tests predictive of therapeutic activity against Alzheimer’s disease

#### Effect of SAR502250 on Aβ_25–35_-induced cell death in rat embryonic neurons

Male and female E18 Wistar rats were used. Embryonic hippocampal cells were cultured in neurobasal medium (Thermo Fisher Scientific) supplemented with glutamine (Thermo Fisher Scientific) and B27 supplement (Thermo Fisher Scientific) for 6 days. Cells were treated with drugs (LiCl or SAR502250) and Aβ_25–35_ (BACHEM) for 36 hours. After the incubation period, cell viability was measured by MTS assay (Promega). Statistical differences between the groups were determined by Dunnett’s multiple comparison test.

#### Effect of SAR502250 on tau hyperphosphorylation (S_396_) *in vivo* in P301L human tau transgenic mice

Three-month-old female P301L human tau transgenic mice (JNPL3), having an average weight of 32 g at the time of testing were used. They received a single dose of SAR502250 (1, 3, 10, 30 and 100 mg/kg/d) by oral route. One hour after the administration, brains and spinal cords were rapidly dissected and quickly frozen. Tissue was homogenized with homogenization buffer (62.5 mM Tris-HCl pH 6.8, 2.3% SDS, 1 mM EDTA, 1 mM EGTA, 1 mM DTT, Protease inhibitor cocktail (Sigma-Aldrich), Phosphatase inhibitor cocktail (Roche Diagnostics). Homogenized sample was boiled for 5 min and centrifuged at 15,000 x g for 15 min. Supernatant was collected and protein concentration was measured by DC protein assay (Bio Rad). 10 μg of samples were applied on 10% SDS-PAGE and transferred onto nitrocellulose membranes. Total human tau protein and phosphorylated (S_396_) tau protein was evaluated by western-blotting labelling with TauN (BD Transduction) and PS396 (Thermo Fisher Scientific) antibodies respectively. Each band was visualized with ECL kit (Amersham Bioscience) and detected with LAS 1000 (Fuji Film).

#### Effects of SAR502250 on short-term visual episodic memory deficit following the central infusion of Aβ_25–35_ peptide using the object recognition test (ORT) in mice

Male Swiss mice weighing 20–22 g, 4–5-week-old at the beginning of the experiment were used. The procedure was the same as described by Griebel *et al*.^30^ and was based on that originally described by Ennaceur and Delacour^31^ in rats and adapted for use in mice. The apparatus consisted of a uniformly lit (20 lux) PVC enclosure (52 L × 52 W × 40 H cm) with a video camera positioned 160 cm above the bench. The observer was located in an adjacent room fitted with a video monitoring system. The experiment consisted of 3 sessions. During the first session (context habituation), the subjects were allowed 2 min to become acquainted with the apparatus. Time in active locomotion was manually recorded with a precision of ±0.1 sec. The animals were again placed in the enclosure 24 hours thereafter for the second (acquisition) session, during which they were exposed to a pair of identical objects (5.5 L × 2 L × 3.3 H cm grey metal triangle or 3 L × 3 W × 3 H cm plastic pyramid) placed 10 cm away from the 2 opposite corners of the back wall.

Animals were left in the enclosure for the amount of time necessary to spend at most 15 seconds exploring these 2 objects within a 5 min timeframe. Animals were removed from the cage once they had reached the 15-second exploration time. Exploration of an object was defined as the animal having its head within 1 cm of the object while looking at it, sniffing it or touching it. Any animal spending less than 15 seconds exploring the 2 objects within 5 min was eliminated from the study. Two different sets of objects were used to allow for cleaning between 2 consecutive animals in order to minimize olfactory cueing. Combinations of orders of presentation and locations of objects were counterbalanced to reduce potential biases owing to spatial or object preferences. During the third (recall) session, animals were exposed to the familiar (i.e. presented during the previous acquisition session) and a novel (i.e. never presented before) object for 4 min, and the time spent exploring each object was recorded. Any animal spending less than 3 seconds exploring both objects was discarded from the study. This third session took place one hour following the second session.

The peptide solution (BACHEM, Switzerland) was incubated at 37 °C for 4 days prior to the administration. 3 µL of the Aβ_25–35_ solution (9 nM of peptide) were slowly injected into the lateral ventricle of isoflurane-anesthetised mice. Injection was done manually, without the help of a stereotaxic apparatus. Accuracy of the injection was checked in a preliminary experiment using indian ink and reached a 95% confidence. The control peptide (scrambled Aβ_25–35_) consisted of the same sequence of amino acids, but in a random order. It was prepared and administered following the same procedure. Ten days after Aβ_25–35_ administration, animals were tested in the ORT. SAR502250 was injected acutely, p.o. 60 min before the acquisition session. The data were expressed as ratio “new/(familiar + new) × 100”, representing the recognition index (RI), and analysed using a Kruskal-Wallis multiple comparisons test. Eight to ten animals per group were used.

#### Effects of SAR502250 on short-term visual episodic memory deficit in APP (SW)/Tau (VLW) mice using the ORT

The procedure was the same as described by Griebel *et al*.^30^. Six-month-old male APP (SW)/Tau (VLW) mice and their non-transgenic wild-type littermates were used for the study. Both were backcrossed to C57BL/6J mice and then crossed together to generate double transgenic mice co-expressing both transgenes on a C57BL/6J genetic background. The experimental setup is based on that described in the previous paragraph with slight modifications. Briefly, in the first session, mice were allowed to become familiar with the experimental environment for 2 minutes. Time spent in activity was measured. Twenty-four hours later, mice were again placed in the enclosure in the presence of two identical objects until they had explored them for a total duration of 15 s. After a forgetting interval of 60 min, mice were placed again in the enclosure with a previously presented object and a new object for 4 min. Time spent exploring the familiar and the new objects were recorded. SAR502250 was administered orally once-a-day for 7 weeks. The last administration was given 30 minutes prior to the second session (i.e. acquisition). The RI was analysed using one-way ANOVA using a fixed factor of treatment or genotype and complementary post hoc (Dunnett) tests were performed. Seven to eleven animals per group were used.

#### **Characterization of SAR502250 in tests predictive of therapeutic activity against psychiatric symptoms**

To investigate potential effects of SAR502250 in comorbid symptoms of AD, we used wild-type animals/species, which were found to be more suitable than the pharmacological or transgenic models of AD in the current experimental procedures.

### Depression

#### Effects of SAR502250 in the differential reinforcement of low rate-72s (DRL-72s) procedure in rats

The procedure was the same as described by Louis *et al*.^32^. Male Wistar rats (10-week-old) were singly housed with *ad libitum* access to water except during operant sessions. Their weight was kept at 450 ± 50 g by feeding with 20 g of food chow given at the end of the day and over the weekend. The experiments were carried out in eight identical rat operant chambers (Med Associates, East Fairfield, VT, USA), each fitted with a 2.8 W overhead house light and a stainless-steel rods floor. A 4.8 × 1.9 cm lever was positioned on the right side of a food tray, which was connected to a food pellets (45 mg, Formula P, Noyes, Research Diets, New Jersey, USA) dispenser. Each operant chamber was enclosed in a ventilated and sound-attenuating cubicle; all events were recorded and controlled by the ‘Med-PC’ software.

Acquisition of the Operant Behavior: Rats were first trained (5 days a week) in daily 30 min sessions to press a lever to obtain a food pellet under a continuous reinforcement-fixed time 60 s concurrent schedule (i.e. if the rat did not press the lever within 60 s, a reinforcement was automatically delivered). When rats obtained at least 100 pellets per training session, they were subjected to a differential reinforcement of low-rate (DRL) 15 s schedule. More explicitly, a lever-press occurring before a delay of 15 s had elapsed was not rewarded and the timer was reset to 0 s for a further 15 s cycle. Session duration of these DRL sessions was set to 60 min. Across successive DRL sessions, the timing of the DRL schedule was progressively increased from 15 to 30 s to the final timing of 72 s. In order to acquaint them to the i.p. injection procedure, rats were injected with saline 30 min pre-session once they attained the DRL-72 s stage. Once performance had stabilized (ie less than 10% variation of total number of responses during six consecutive DRL-72 s vehicle sessions, and less than seven reinforcers per session), rats were subjected to pharmacological challenge sessions. Each rat received three drug treatments, with doses administered in a mixed order. For a given drug treatment, control values were calculated by averaging the performance of all vehicle sessions immediately preceding all drug sessions. Furthermore, a stability criterion (less than 10% variation of total number of responses between the vehicle session immediately before the drug session and the six vehicle sessions preceding the start of the pharmacological study: vide supra) was in effect in-between each drug session. Fluoxetine or the appropriate vehicle was administered i.p. 30 min pre-session, and SAR502250 was administered p.o. 60 min pre-session.

Data: The following parameters were automatically recorded by the Med-PC software: the total number of lever-presses emitted during the session, the number of food pellets obtained (i.e. the number of reinforced responses), and the inter-response time (IRT, the time elapsed between two lever-presses^33^). IRTs were subsequently split into nine bins (IRT bin (0–12 s), IRT bin (13–24 s), IRT bin (85–96 s), and IRT bin ( 49-96s)). From these raw data, the percentage of lever-presses emitted in each of the nine 12 s bins and the percentage of lever-presses reinforced were calculated using a Macro procedure with the Excel software (percentages were calculated as a function of the total number of lever-presses emitted during the session). The percentages of lever-presses during two IRT bins in particular were analyzed: (1) the IRT bin (0–12 s), corresponding to the ‘burst responses’ and shown to be sensitive to benzodiazepine-like compounds^33^; (2) the IRT bin (49–96 s), as it has been shown to be particularly sensitive to antidepressant drugs^34^.

Statistical analysis: The percentage of lever-presses emitted in bins (0–12 s) and (49–96 s), the percentage of lever-presses reinforced, and the total number of lever-presses were analyzed with a Friedman’s test, followed by post hoc tests with a Dunn’s correction factor for a comparison between drug and control (vehicle-treated) groups. Statistical analyses were performed with the SAS system 8.2 (SAS Institute Inc., Cary, NC, USA). Eight animals per group were used

#### Effects of SAR502250 in the chronic mild stress (CMS) test in mice

Male BALB/c mice weighing 24–29 g and 5-6-week-old at the beginning of the experiment were used. Earlier studies demonstrated that this strain is particularly suitable for investigating the antidepressant-like effects of drugs in this model^35^. The CMS protocol is based on that used by Griebel *et al*.^36^ and consists of the sequential application of a variety of mild stressors, including restraint, forced swimming in warm (35 °C) water, water and/or food deprivation, pairing with another stressed animal, each for a period of between 2 and 24 hours. The CMS procedure lasted 43 days. The physical state was measured according to a physical state scale attributing 3 points to well-groomed and clean animals, 2 points to animals with disorganized coat and 1 point to animals showing loss of fur and dirty fur, once-a-week over the 42-day CMS period. The administration of SAR502250 (30 mg/kg, i.p., once-a-day), fluoxetine (10 mg/kg, i.p., once-a-day) or lithium chloride (100 mg/kg, i.p., once-a-day) started 15 days after the beginning of the stress exposure and lasted until the CMS was completed (in total, 28 days of treatment).

Physical state data (expressed on a physical state scale from 1 to 3) from control stressed and unstressed animals were analyzed for each week of the CMS by a Wilcoxon test. Subsequently, physical state data from the 3 groups of stressed/treated animals were compared using a Kruskal-Wallis test followed by Kruskal-Wallis one-sided upper multiple comparisons tests with Bonferroni-Holm correction versus stressed control group (as increases in physical data scores are expected for mice treated with either fluoxetine, lithium chloride or SAR502250). Twenty animals per group were used.

### Anxiety

#### Effects of SAR502250 in the mouse defense test battery (MTDB)

Ten-week-old male OF1 mice weighing 40–55 g were used. Previous studies showed that this strain is particularly suitable for investigating the anxiolytic-like effects of drugs in this model^37^. The test was conducted in an oval runway as described by Griebel *et al*.^38^. Pretest: Sixty minutes after p.o. administration of SAR502250 (10, 30 and 100 mg/kg), lithium chloride (50, 100 and 200 mg/kg) and diazepam (1, 3 and 10 mg/kg), the mouse was placed into the runway for a 3-min familiarization period, in which locomotor activity (number of line crossings) was recorded. The rat avoidance test: Immediately after the 3-min familiarization period, the experimenter introduced a hand-held dead male Long Evans rat (370–375 g, 10-week-old, killed by CO2 inhalation just before the beginning of the experiment) 5 times at one end of the runway and brought up to the mouse at a speed of approximately 0.5 m/s. Approach was terminated when contact with the mouse was made or the mouse ran away from the approaching rat. Flight was measured by the number of avoidances of 5 trials. Chase/flight test: The rat was then brought up to the mouse at a speed of approximately 2 m/s. A constant distance of 2 meters separated the rat and the mouse when the rat was introduced in the runway. Risk assessment was given by a measure of the number of stops (pauses in movement). The rat was removed after the chase was completed. Forced contact in the straight alley: By closing 2 doors (60 cm distant from each other), the runway was then converted to a straight alley in which the mouse was confined. The experimenter brought the rat into contact with the mouse in the straight alley. Approaches were directed quickly (within 1 second) towards the rat’s head. For each such contact, defensive aggression was measured by the number of bites by the mouse to the rat. Data concerning locomotor activity (number of line crossings), flight (number of avoidances), risk assessment (number of stops) and defensive aggression (number of bites) were subjected to analysis. Data were either assessed using a Student’s t-test (diazepam) or a one-way ANOVA (SAR502250), or with the non-parametric tests, Wilcoxon (diazepam) or Kruskal-Wallis followed in case of significant effects by post-hoc one-sided lower Kruskal-Wallis multiple comparison tests versus respective control groups, as decreases in the different variables (number of avoidances, stops and bites) were expected for treated groups (SAR502250). Eight to eleven animals per group were used

### Agitation

#### Effects of SAR502250 on hyperactivity induced by amphetamine in mice or by PCP in PCP-sensitized rat

In the first experiment, Male Swiss mice (22–26 g, 5–6-week-old) were orally pretreated with SAR502250 (10, 30 and 60 mg/kg) or vehicle, followed 30 min later by a challenge administration of vehicle or amphetamine at 2 mg/kg, i.p. Immediately thereafter, they were placed in the activity cages devices (20 cm diameter, 9.5 cm height, Apelex, France) and locomotor activity was recorded for a period of 30 min. In the second experiment, Male Sprague-Dawley rats (80–100 g, 5-week-old) were administered PCP at 10 mg/kg, i.p. for 5 consecutive days. Three days later, they were habituated to the activity cages (38 × 38 × 25 cm high) for 60 minutes, before receiving an oral administration of SAR502250 (3, 10 and 30 mg/kg). They were immediately replaced in the activity cage for further 30 minutes (in order to test for potential motor side-effects of SAR502250), and finally they received an acute challenge dose of PCP at 1.5 mg/kg, i.p. before being replaced in the activity cages for a last 30 minutes period. In summary, locomotor activity was recorded for 2 hours (habituation 60 min + treatment-period 30 min + post-challenge period 30 min). Statistical analyses were performed using SAS V8.2 software (SAS Institute, Cary, NC, USA). The number of light beam breaks (motility count) recorded were analyzed using one-way ANOVAs using a fixed factor of challenge or genotype and complementary post hoc (Newman-Keuls) tests were performed. Nine to ten animals per group were used.

### Sensorimotor gating deficit

#### Prepulse inhibition (PPI) deficit in Wistar rats

Male Wistar Rats (260–300 g, 7–8-week-old) were tested in startle boxes (Med Associates, East Fairfield, VT, USA). The startle reflex was detected via a piezoelectric transducer situated below the startle platform and recorded via a computer. Rats were placed into a restraint cylinder (8 × 19 cm) fixed on top of the startle platform. On the first day of the experiment, they were evaluated for their spontaneous PPI to distribute rats across the groups with similar-relatively low levels of PPI before pharmacological testing performed a day after. In order to test PPI abilities, rats were subjected to an initial habituation session that started with a 5-min adaptation period with a background white noise of 65 dB, followed by five pulse stimuli to accustom the rats to the startling pulses (120 db, 50 ms duration, inter-pulse 10 to 15 seconds), but these startling responses were excluded from the data analysis. Then, rats were submitted to a session of 40 trials of randomized paired “Pre-Pulse (20 ms duration: 65; 72; 79; 85 dB) – Pulse (120 dB, 50 ms duration)” stimuli with a 40 ms of time delay preceding the pulse and with an inter-trial time (null period) of 10 to 15 seconds. During the null period, there was no presentation of acoustic stimulus with the exception of the background noise (65 dB). Prepulse inhibition was calculated for each rat, from averaged startle amplitudes for each of the three prepulse intensities across all test trials. The percentage of pre-pulse inhibition (expressed as percentage) was calculated as follows: (Startle amplitude following pulse − Startle amplitude following prepulse) *100/ Startle amplitude following pulse = % of PPI. On day 2, for pharmacological studies, rats were subjected to the same procedure as day 1 but were distributed (Latin square randomization) in five pharmacological distinct groups, in order to obtain similar average levels of PPI between groups, before testing. Each group was tested with either vehicle, clozapine (10 mg/kg) or SAR502250 (3, 10 and 30 mg/kg), which was administered orally 60 min prior to testing. Sixteen to nineteen animals per group were used. The data were analyzed with a Kruskal-Wallis test followed in case of significant effects by post-hoc Kruskal-Wallis multiple comparison tests versus respective control groups. For additional details on the procedure, see^39^.

## Results

### Characterization of SAR502250 in tests predictive of therapeutic activity against Alzheimer’s disease

#### Effect of SAR502250 on Aβ_25–35_-induced cell death in rat embryonic neurons

**Figure 2:**
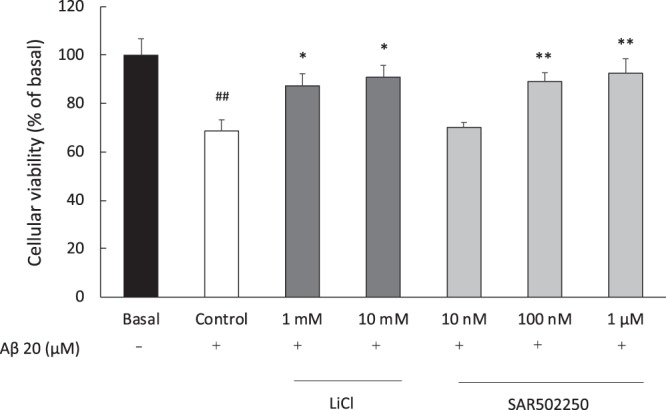
Effects of increasing concentrations of SAR502250 and lithium on Aβ_25–35_-induced cell death in rat embryonic hippocampal neurons. Cells were treated with the experimental drugs and Aβ_25–35_ for 36 hours. Data represent mean ± SEM. ^##^P < 0.01 (vs Basal, t-test); *P < 0.05, **P < 0.01 (vs Control, Dunnett). N = 6.

The β-amyloid fragment Aβ_25–35_ (20 μM) significantly increased cell death in rat embryonic hippocampal neurons (t-test: P < 0.01). This effect was attenuated significantly by SAR502250 at 100 nM and 1 μM (P < 0.01), and by lithium at 1 and 10 mM (P < 0.05) (Fig. 2).

#### Effect of SAR502250 on tau phosphorylation (S_396_) *in vivo* in P301L human tau transgenic mice

**Figure 3:**
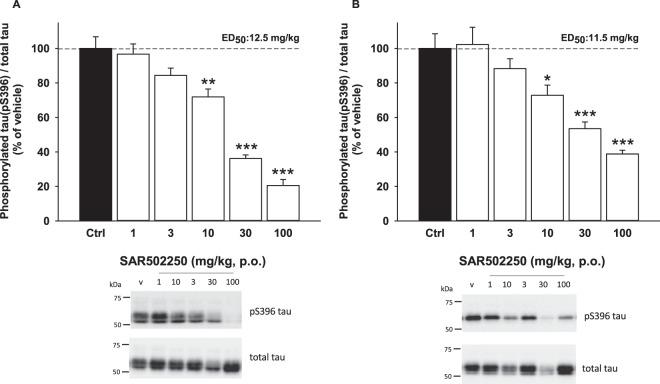
Effect of increasing doses of SAR502250 on tau hyperphosphorylation (S_396_) in the cortex (**A**) and spinal cord (**B**) of P301L human tau transgenic mice. Time of administration was 60 minutes. Data represent mean + SEM. *P < 0.05, **P < 0.01 and ***P < 0.01 (vs Ctrl, Kruskal-Wallis). Blots cropped from different parts of the same gel are shown. Full-length blots are shown in Fig. S1. N = 6.

SAR502250 attenuated dose-dependently tau phosphorylation in the cortex [F(5,30) = 47,35, P < 0.0001; Fig. 3A] and spinal cord [F(5,30) = 15.41, P < 0.0001; Fig. 3B] of transgenic mice expressing P301L tau. Post-hoc statistical analyses showed that these effects were significant from 10 mg/kg in both structures, with ED_50s_ of 12.5 and 11.5 mg/kg, respectively.

#### Effects of SAR502250 on short-term visual episodic memory deficit following the central infusion of Aβ_25–35_ peptide using the object recognition test (ORT) in mice

**Figure 4:**
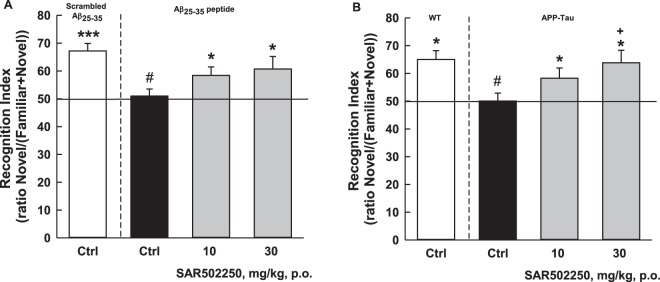
Effects of SAR502250 on short-term visual episodic memory deficit following the central infusion of Aβ_25–35_ peptide in Swiss mice. The vehicle or the drug was given 60 min before the acquisition session (**A**) or in APP (SW)/Tau (VLW) mice (**B**) using the novel object recognition test. The vehicle or the drug was administered once-a-day for 7 weeks. The last administration was given 30 minutes prior to the second session. Data are expressed as recognition index + SEM. *P < 0.05 and ***P < 0.001 (vs. chance level); ^#^P < 0.05 (vs. Scrambled or WT); ^+^P < 0.05 (vs. Ctrl APP-Tau) (Kruskal-Wallis or Dunnett). N = 7–11.

Mice that received the scrambled Aβ_25–35_ peptide spent more time investigating the novel object [17.61 vs 8.77 s, recognition index (RI) = 67.2; Fig. 4A], an effect which was abolished by the infusion of Aβ_25–35_ [10.26 vs 10.15 s, RI = 51]. SAR502250, administered at 10 and 30 mg/kg before the acquisition session, significantly (χ^2^ = 6.72, P = 0.03) restored this preferential investigation [10 mg/kg: 13 vs 8.59 s, RI = 58.41; 30 mg/kg: 12.79 vs 8.26 s, RI = 60.74] (Fig. 4A).

#### Effects of SAR502250 on short-term visual episodic memory deficit in APP (SW)/Tau (VLW) mice using the ORT

Wild-type mice spent more time investigating the novel object [8.4 vs 4.8 s, RI = 65.02; Fig. 4B]. This preference for the novel object was not observed in APP (SW)/Tau (VLW) mice [8.4 vs 8.4 s, RI = 50.07]. SAR502250, administered to transgenic animals at 10 and 30 mg/kg for 7 weeks, significantly [F(3,55) = 3.64, P = 0.018] restored this preferential investigation [10 mg/kg: 9.1 vs 6.7 s, RI = 58.29; 30 mg/kg: 9.6 vs 5.9 s, RI = 63.82] (Fig. 4B).

### Characterization of SAR502250 in tests predictive of therapeutic activity against psychiatric symptoms

#### Depression

Effects of SAR502250 in the differential reinforcement of low rate-72s (DRL-72s) procedure in rats: The prototypical antidepressant, fluoxetine at 10 mg/kg, increased significantly (Friedman’s H(3) = 11, P = 0.012) percentage of lever-presses in the 49–96 s IRT, leading to a significant [H(3) = 8.60, P = 0.036] augmentation of the percentage of reinforced responses. The drug did not modify the percentage of ‘burst responses’ [i.e. percentage of responses in the IRT bin (0–12 s)] [H(3) = 4, P = 0.26]. Moreover, the antidepressant significantly [H(3) = 13.20, P = 0.004] reduced the total number of responses (Table 1). Similarly, SAR502250 (10 mg/kg, i.p.), significantly [H(2) = 9.75, P = 0.008] increased the percentage of lever-presses in the IRT bin (49–96 s), with a significant [H(2) = 6.25, P = 0.04] augmentation of the percentage of reinforced responses. SAR502250 did not affect significantly the percentage of ‘burst responses’ [H(2) = 0.75, P = 0.69] or the total number of responses [H(2) = 5.25, P = 0.07] (Table 1).

**Table 1:** Effects of fluoxetine and SAR502250 on the percentage of responses emitted in the IRT bins (49–96 s) and (0–12 s), on the percentage of reinforced lever presses, and on the total number of lever presses in the differential reinforcement of low rate-72s (DRL-72s) procedure in rats.

Drugs	Doses (mg/kg)	Percentage bin (48–96 s)	Percentage reinforced presses	Percentage bin (0–12 s)	Total presses
Fluoxetine	0	17.29 ± 3.38	2.80 ± 0.60	17.03 ± 2.58	117.72 ± 8.80
(i.p.)	2.5	19.36 ± 3.87	4.47 ± 1.13	15.87 ± 1.26	109.33 ± 7.78
	5	23.23 ± 4.44	4.98 ± 0.99	14.86 ± 2.11	107.17 ± 8.92
	10	38.07 ± 5.52*	12.40 ± 3.68*	11.53 ± 3.09	83.33 ± 6.01*
SAR502250	0	16.42 ± 2.66	1.96 ± 0.28	12.68 ± 1.29	114.44 ± 4.49
(p.o.)	10	26.21 ± 4.47*	4.50 ± 1.22	11.09 ± 2.01	101.75 ± 5.17
	30	29.42 ± 3.25**	6.10 ± 1.12*	11.55 ± 1.40	96.75 ± 4.28

Fluoxetine or the appropriate vehicle was administered 30 min pre-session, and SAR502250 was administered 60 min pre-session. *P < 0.05, **P < 0.01 vs vehicle-treated group (0): post hoc tests with a Dunn’s correction, following a significant Friedman analysis. N = 8 rats per drug treatment.

**Figure 5:**
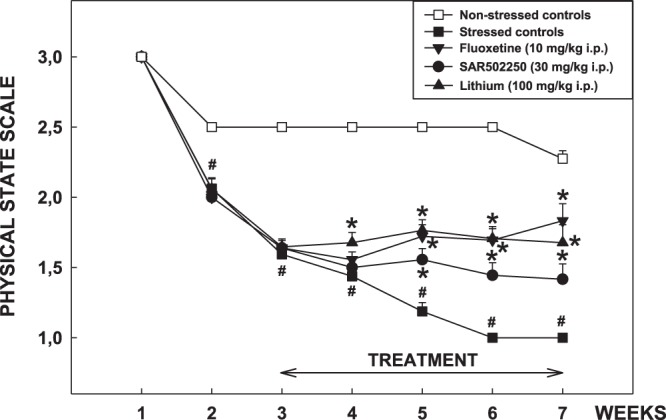
Effect of SAR502250, fluoxetine and lithium in the chronic mild stress procedure in mice. The vehicle or the drugs were given once-a-day 15 days after the beginning of the stress exposure and lasted until the CMS was completed. Data are expressed as mean physical state score + SEM. #P < 0.01 (Wilcoxon test versus non-stressed control group), *P < 0.05, **P < 0.001 (Kruskal-Wallis one sided upper multiple comparison tests with Bonferroni-Holm correction versus stressed control group). N = 20 mice per group.

Effects of SAR502250 in the chronic mild stress (CMS) test in mice: Results indicated a significant stress-induced degradation in the physical state of the coat from week 2 to week 7 (Wilcoxon: at least P < 0.05 for each week) (Fig. 5). This effect was improved by SAR502250 at 30 mg/kg from week 5, by fluoxetine at 10 mg/kg from week 4 and by lithium from week 5. All these effects lasted until the end of CMS (Fig. 5).

#### Anxiety

**Figure 6:**
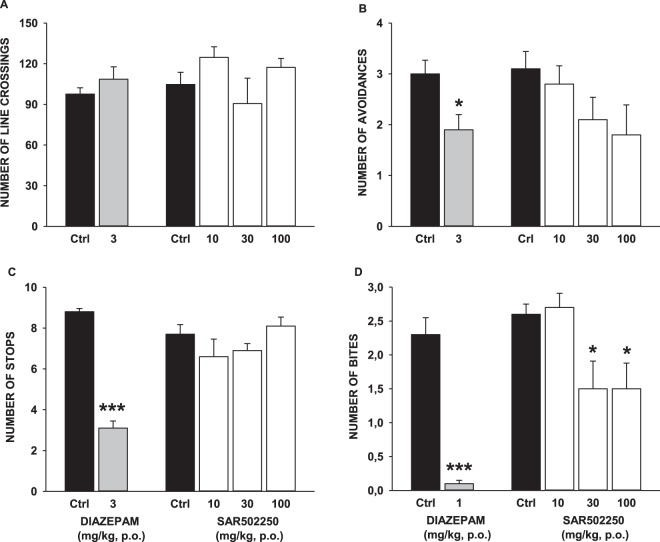
Effects of SAR502250 and diazepam in the mouse defense test battery on (**A**), locomotor activity prior to the exposure to the threat; (**B**), flight response in response to the approaching rat; (**C**), risk assessment when the rat was chasing the mouse, and (**D**), defensive attack reactions upon forced contact with the rat. The vehicle or the drugs were administered 60 minutes prior to testing. Data represent mean + SEM, *P < 0.05 and ***P < 0.001. N = 8–11 mice per group.

Effects of SAR502250 in the mouse defense test battery (MTDB): Before mice were exposed to the threat stimulus, neither SAR502250 [ANOVA: F(3,36) = 3.77, P = 0.02; post-hoc analysis vs. control: P > 0.05] nor diazepam (Student: t = 1.07, P = 0.30) significantly modified line crossings. In the avoidance phase, diazepam (Wilcoxon: S = 46, P = 0.002), but not SAR502250 (χ^2^ = 5.36, P = 0.15) significantly decreased avoidance frequency. When mice were chased by the threat stimulus, diazepam (S = 36, P = 0.0002), but not SAR502250 [F(3,36) = 0.08, P = 0.97] reduced significantly stops. Upon forced contact with the threat stimulus, SAR502250 significantly reduced the frequency of bites (χ^2^ = 9.91, P = 0.0.02) at 30 mg/kg, and diazepam at 3 mg/kg (S = 36, P = 0.0002). The data are shown in Fig. 6.

#### Agitation

**Figure 7:**
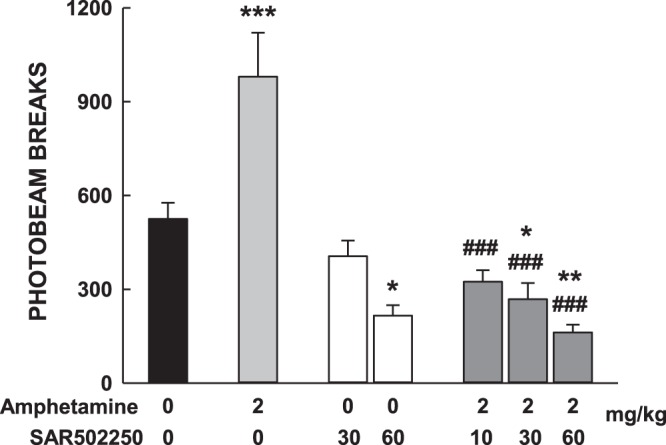
Effects of SAR502250 on motor hyperactivity induced by amphetamine in mice. Animals were pretreated with SAR502250 or vehicle, followed 30 min later by a challenge administration of vehicle or amphetamine. Immediately thereafter, they were placed in the activity cages devices. Data represent mean + SEM, *P < 0.05, **P < 0.01 and ***P < 0.001 (vs vehicle control); ^###^P < 0.001 (vs amphetamine, Newman-Keuls). N = 10–11 mice per group.

Effects of SAR502250 on hyperactivity induced by amphetamine in mice or by PCP in PCP-sensitized rat: Results indicated that animals treated with amphetamine displayed a significant increase in locomotor activity compared to rats that received vehicle [ANOVA: F(6,64) = 17.51, P < 0.0001]. This effect was prevented by SAR502250 at all doses (10, 30 and 60 mg/kg). SAR502250 alone at the highest dose or in combination with amphetamine at 30 and 60 mg/kg significantly reduced activity compared to rats treated with vehicle (Fig. 7).

**Figure 8:**
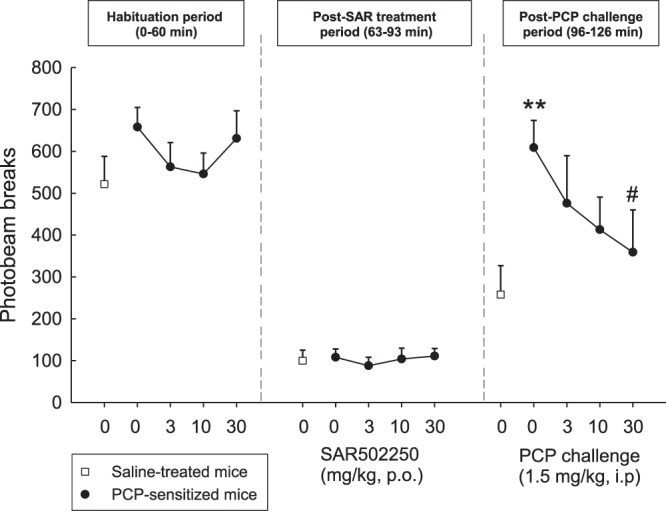
Antagonism by SAR502250 of the hypersensitivity to an acute challenge with PCP in rats sensitized to PCP. Data represent mean + SEM number of infrared beam interruptions recorded for 90 min, immediately after an injection of PCP (1.5 mg/kg i.p.) or vehicle, which was preceded 30 min earlier by an p.o. injection of SAR502250 or vehicle. **P < 0.01, PCP-sensitized rats compared to vehicle-treated rat. ^#^P < 0.05 compared to acute PCP/PCP-sensitized rats. N = 9–10 rats per group.

In the PCP experiment, the psychotomimetic produced a 159% increase in the number of infrared beams interruptions in control rats (t = 2.66, P = 0.03). This increase was more than doubled (+136%) in PCP-treated rats (t = 8.44, P < 0.0001), indicating an hypersensitivity to the locomotor-stimulating effects of acute PCP. SAR502250 dose-dependently attenuated the hypersensitivity, producing an almost complete antagonism at 30 mg/kg. This effect is displayed in Fig. 8, showing the dose-dependent attenuation of SAR502250 on the differential sensitivity to PCP between PCP-sensitized and control rats. The attenuating effect of SAR502250 reaching statistical significance (χ^2^ = 9.12, P = 0.058) with a significant effect of SAR502250 at 30 mg/kg (P = 0.02). The doses of SAR502250 preventing hypersensitivity to PCP in PCP-treated rats, had no effect when given alone on spontaneous motor activity (recorded during the thirty minutes prior to the administration of PCP (χ^2^ = 1.26, P = 0.87). This suggests a selective action of SAR502250 against PCP-induced hypersensitivity, and not a non-specific locomotor effect. Finally, basal activity (recorded during the habituation period) for vehicle and PCP groups did not differ statistically [F(4,42) = 1.02, P = 0.41].

#### Sensory gating deficit

**Figure 9:**
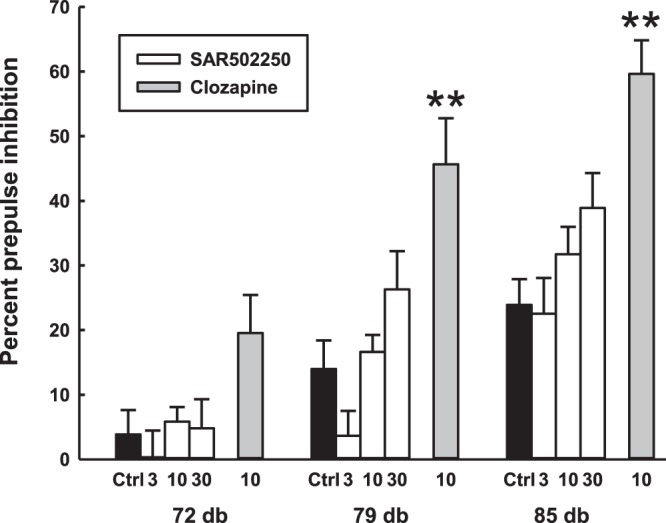
Reversion by SAR502250 and clozapine of a spontaneous deficit of prepulse inhibition of the startle reflex in Wistar rats. Prepulse inhibition expressed as a function of prepulse (PP) intensity (in dB). The vehicle or the drugs were administered 60 minutes prior to testing. Each bar represents the mean + SEM. *P < 0.05, **P < 0.01 vs vehicle (Kruskal-Wallis or Wilcoxon test at the corresponding prepulse intensity. N = 16–19 rats per group.

Prepulse inhibition (PPI) deficit in Wistar rats: Wistar rats were shown to have PPI baseline level, ranging in control animals between 3.85 ± 3.78% and 23.90 ± 3.96% depending of the intensity of the acoustic prepulse that was applied (Fig. 9). Kruskal-Wallis analyzes indicated a significant effect of treatment on spontaneous PPI in Wistar rats at 79 (χ^2^ = 25.72, P < 0.0001) and 85 (χ^2^ = 23.86, P < 0.0001), but not at 72 dB (χ^2^ = 7.04, P = 0.13). SAR502250 (30 mg/kg) enhanced PPI at the intensity of 85 dB, but the effect failed to reach statistical significance (P = 0.10). This was in contrast to clozapine, which significantly potentiated PPI at 79 and 85 dB (both P < 0.001). Basal startle amplitude was not significantly altered by SAR502250 or clozapine (data not shown).

## Discussion

This work had two main goals. The first was to evaluate the neuroprotective potential of the GSK3 inhibitor, SAR502250. The second objective was to verify whether the drug is able to attenuate behavioral alterations reminiscent of NPS associated with AD. Results demonstrated that the drug reduced tau hyperphosphorylation, neuronal cell death and ameliorated some NPS.

### Characterization of SAR502250 in tests relevant to assess therapeutic activity against AD

There is evidence indicating that GSK3 is closely involved in tau hyperphosphorylation, the increased production of Aβ in AD pathology and in memory impairment^7,40^. The kinase induces hyperphosphorylation of tau at several primed (e.g., Tyr231) and non-primed (e.g., S_396_) phosphorylation sites in cellular neurodegeneration assays, indicating that GSK3 is a key tau kinase in the formation of neurofibrillary tangles (NFT) and ultimately neuronal death^8^. Consistent with these findings, conditional transgenic mice overexpressing GSK3 display tau hyperphosphorylation and neurodegeneration^41^, while pharmacological inhibition of the kinase prevents tau hyperphosphorylation in normal mice or in a transgenic model of AD^28,29,42–45^. In line with these findings are the present data with SAR502250 demonstrating that oral administration of the drug decreased hyperphosphorylation on S_396_ in the spinal cord and cortex in P301L human tau transgenic mice.

There is accumulating evidence that GSK3 plays a role in the function of Aβ, which is further upstream of tau in the pathological progression of AD^46^. Exposure of neuronal cells to toxic Aβ increases the activity of GSK3, which in turn facilitates the processing of amyloid precursor protein (APP) leading to an upregulation of Aβ synthesis and aggregation via the phosphorylation of tau. Genetic or pharmacological deactivation of GSK3 was reported to decrease toxicity associated with Aβ, ameliorate Aβ-induced behavioral impairments, and rescue the loss of neurons in APP-overexpressing mice^11,47–53^. The results of our study are in line with these observations. They showed that SAR502250 prevented the increase in cell death in rat embryonic hippocampal neurons following application of the neurotoxic fragment of the full-length Aβ peptide, Aβ_25–35_. The intra ventricle injection of the Aβ_25–35_ peptide was demonstrated to produce neurotoxic effects similar to those produced by the Aβ_1–40_ peptide^54,55^, including cellular and cerebral oxidative stress, and neuroinflammation, modified endogenous amyloid processing, provoking hippocampal morphological alterations and a rapid glial activation^56^. Moreover, studies demonstrated impairment in memory processes when the amyloid fragment was infused into the ventricle or locally applied into the hippocampus of rodents^56–62^. In this study, infusion of the β_25–35_ fragment in cerebral ventricles of mice produced a deficit in short-term episodic memory in the object recognition test. This effect can be attributed to Aβ_25–35_, since the scrambled Aβ_25–35_ peptide had no such an action. The administration of SAR502250 prior to the acquisition session attenuated this deficit. The procognitive effects of SAR502250 were demonstrated further in transgenic APP(SW)/Tau(VLW) mice, described to display many pathological features, reminiscent of those observed in AD, including severe learning deficit^63,64^. Here, they displayed impaired memory performance in the object recognition task, which were completely rescued by SAR502250 following a 7-week treatment. The memory-improving effects of SAR502250 agree with previous studies showing that other GSK3 inhibitors enhance cognitive functions in animal models^65–68^. The ability of GSK3 inhibitors to improve memory performance has been suggested to involve various mechanisms, including modification in synaptic plasticity (i.e. facilitation of long-term potentiation (LTP) induction and long-term depression diminution) and promoting adult hippocampal neurogenesis^69^. Altogether, the current findings with SAR502250 in experimental models of AD support further the idea that GSK3 inhibition represents a potential disease-modifying approach for the treatment of this condition. Although several GSK3 inhibitors have been in the pipeline for the treatment of AD, proof of efficacy has not been established yet. A double-blind, placebo controlled, Phase 2 study with the GSK3 inhibitor tideglusib in mild to moderate AD patients did not show clinical efficacy, although mild AD participants in the lowest dose group showed significant responses on the primary and several secondary measures of efficacy, suggesting that disease stage and GSK3 inhibition level are critical parameters in clinical studies with GSK3 inhibitors in AD patients^70^.

### Characterization of SAR502250 in tests of psychiatric symptoms

It is widely acknowledged that AD presents with a complex clinical phenotype including several psychiatric symptoms, some of which predict disease prognosis. Unfortunately, their clinical management remains challenging because AD patients respond poorly to existing medications. Depression is among the most prevalent psychiatric symptom in AD affecting 42% of patients based on a recent meta-analysis^2^. GSK3 inhibitors have been demonstrated to produce antidepressant-like effects. Kaidanovich-Beilin and colleagues were the first to demonstrate that L803-mts, a peptide inhibitor of GSK3, produces antidepressant-like effects in the forced-swimming test following intracerebral ventricle injections in mice^24^. Follow-up studies using the same behavioral assay extended these findings, showing antidepressant-like effects after systemic administration of other GSK3 inhibitors, such as AR-A014418^25,71^ and NP031115^26^. More recently, repeated administration of the GSK3 inhibitor VP2.51 was found to produce antidepressant-like effects in the forced-swimming test in non-stressed mice as well as in previously stressed animals^27^. While the forced-swimming test is a commonly used assay, it has limited validity as a model of depression^72^. Here, we used the CMS and DRL-72 s schedule procedures, two models claimed to have good face and predictive validity^73,74^. In the DRL-72 s procedure, SAR502250 displayed antidepressant-like activity, increasing the percentage of responses in the inter-response time (IRT) bin (49–96 s), resulting in a higher number of reinforced presses. These effects resemble that elicited in the current study by fluoxetine, a conventional antidepressant, which was used as positive control. However, the magnitude of the effect was less with the GSK3 inhibitor. The antidepressant-like potential of SAR502250 was demonstrated further using the CMS test after chronic administration of the drug for 28 days. SAR502250 ameliorated chronic stress-induced degradation of the physical state of the coat, suggesting that the drug normalized grooming, which was impaired by repeated stress. Again, this effect was less pronounced when compared to the antidepressant, fluoxetine. The precise mechanism(s) by which inhibition of GSK3 results in antidepressant-like activity remain(s) to be delineated. It has been proposed recently that GSK3 inhibitors produce their antidepressant-like action by promoting hippocampal neurogenesis, increasing cell proliferation and survival of newborn neurons^27^. Interestingly, this study further showed that pharmacological blockade of neurogenesis impaired the ability of the GSK3 inhibitor to produce antidepressant-like action, indicating that these effects are neurogenesis-dependent.

Little is known on potential effects of GSK inhibition on the modulation of anxiety and aggression, two frequent comorbid symptoms of AD, whose prevalence is estimated around 40%^2^. One study reported that genetic or pharmacological blockade in the activity of GSK3 reduced anxiety-like behaviors in knock-in mice bearing a mutant form of tryptophan hydroxylase 2 (Tph2), the brain 5-HT synthesis enzyme^13^. However, a similar activity was not observed in normal mice following the administration of the selective GSK3 inhibitor, VP2.51^27^. Here we used the mouse defense test battery (MDTB) to investigate potential effects of SAR502250 on emotional behavior. The behaviors displayed by mice in this test have been shown to relate to different aspects of anxiety and defensive aggression, which relate either to affective-orientated defense reactions or to the process of acquiring and analyzing information in the presence of threatening stimuli^75^. Our findings show that SAR502250 weakly modified risk assessment and flight, behaviors that have been demonstrated to be more sensitive to selective 5-HT reuptake inhibitors or to benzodiazepines, i.e., drugs generally used to treat panic and generalized anxiety disorders. However, it decreased significantly defensive aggression, a terminal defense reaction, suggesting that SAR502250 may be useful to attenuate certain forms of aggressive behaviors. The mechanisms underlying the specificity by which SAR502250 exerts anti-aggressive effects remain to be fully elucidated. Findings with Tph2 knock-in mice, which show a dramatic decrease in central 5-HT function may, however, be relevant to his issue. These mice display enhanced aggressive behavior, a phenomenon which is not observed in animals that were additionally haplo-insufficient for GSK3, indicating that reduced GSK3 expression rescued abnormal aggression in Tph2^13^. While it is tempting to speculate that the 5-HT system plays a role in the anti-aggressive effects of SAR502250 in the MDTB, the current study does not provide any evidence supporting this idea. Clearly, more studies are needed to determine more precisely the mechanisms underlying the anti-aggressive effects of SAR502250.

Hyperactivity, including agitation and excessive motor activity, is a frequently occurring sub-syndrome in AD^2^. It is severe enough to produce disability, worsens as disease severity increases, and is predictive of more rapid decline^1^. Several medications, including antipsychotics, anxiolytics or antidepressants, have been used to treat this syndrome, but have shown limited efficacy^1^. Nevertheless, a recent open label case series in patients with AD showed that a low dose of lithium may represent an effective treatment of agitation^76^, suggesting that GSK3 inhibitors may be useful in the management of this symptom in AD patients. In preclinical studies, transgenic mice overexpressing GSK3 have been shown to display enhanced locomotor activity compared to their wild-type counterparts^77^. Moreover, selective GSK3 inhibitors were found to decrease hyperactivity and expression of behavioral sensitization in the mouse following the administration of psychostimulant drugs, such as amphetamine or cocaine^78–81^. In the present study, SAR502250 attenuated hyperactivity induced by amphetamine in naïve mice or following an injection of PCP in PCP-sensitized rats. Psychostimulant-induced hyperactivity has been associated with an enhanced dopaminergic transmission within the nigrostriatal and mesolimbic dopamine pathways^82–85^. Results from an earlier study showed that the ability of the non-selective GSK3 inhibitor, valproic acid, to inhibit hyperactivity induced by amphetamine is associated with its ability to modulate the activity of GSK3 in the frontal cortex and caudate putamen, which receive dopaminergic projections from the substantia nigra and nucleus accumbens, respectively. Moreover, valproic acid increased phosphorylation of S_9_-GSK3 in the caudate putamen, and S_21_ and S_9_ in the frontal cortex, suggesting an inhibitory action of the kinase in these regions^81^. Although these findings highlight the importance of GSK3 in mediating hyperactivity elicited by enhanced dopaminergic transmission, it is unclear whether the same mechanism is involved in hyperactivity in AD patients and the efficacy of lithium in alleviating this symptom.

Deficit in sensorimotor gating is another symptom that has been shown in AD patients^86–90^. Sensorimotor gating is described as a putative neural mechanism that inhibits the processing of extraneous cognitive, sensory and motor information, allowing mental and behavioral integration^91^. In preclinical studies, this phenomenon is classically evaluated by the prepulse inhibition (PPI) of the startle response. APP/PS1 transgenic mice, which are widely used as a model of AD, display impaired PPI, a deficit associated with Aβ neuropathology and memory impairment^92^. Here, we used Wistar rats, a strain which shows a near total lack of PPI, to model sensory gating deficit^93^. Results showed that SAR502250 failed to significantly attenuate the deficit in PPI in Wistar rats. Although evidence suggests that GSK3 is involved in sensorimotor gating^20,21,23,94^, studies that investigated the effects of genetic and/or pharmacological blockade of GSK3 on PPI have yielded inconsistent results. Several authors reported that GSK3 inhibitors rescued the deficit in PPI in mutant mice displaying a schizophrenia- or compulsive-relevant behavioral phenotype^95–97^. However, contradictory evidence has also been reported. Indeed, findings from experiments using genetic or pharmacological manipulations that reduce GSK3 function have shown an attenuation in PPI in C57BL/6J mice, suggesting that GSK3 facilitates sensorimotor gating^98^. The reasons for this apparent discrepancy in the modulation of PPI following GSK3 inhibition remain to be determined. Perhaps it could reflect differences in the degree to which GSK3 function is altered in these models. Thus, the transgenic mice expressing a disease mutation used in the aforementioned studies^95,96^ display altered GSK3 signaling and impaired interplay with key proteins involved in development, such as DISC1 and AKT1, a feature not seen in wild-type strains such as the Wistar line used in the current study. However, more studies are necessary in order to establish the generality of this assumption.

Taken together, the present findings with SAR502250 on behaviors reminiscent of NPS in AD suggest that GSK3 inhibitors may be useful in treating these comorbid symptoms. However, it is important to emphasize that – with the exception of the cognitive tasks - our results were obtained in non-pathological models of AD. It cannot be excluded that the behavioral profiles displayed by wild-type strains following SAR502250 administration may have been different in transgenic or pharmacological models of AD. Clearly, further experiments using procedures that are more suitable than the current ones to be used with animal models of AD are warranted to determine more precisely the potential of GSK3 inhibitors in managing NPS in AD.

## Conclusion

Our study demonstrates that the selective inhibitor of GSK3, SAR502250 displays efficacy on biochemical and behavioral markers of AD, thus confirming the therapeutic potential of GSK3 inhibitors against this condition. Moreover, the current work provides evidence that blockade of this kinase interferes with behaviors impaired in AD, suggesting that GSK3 inhibitors may have both disease-modifying and symptomatic potential in the treatment of AD.

## Supplementary information

Western blot images of total tau and pS396-tau in P301L human tau transgenic mice cortex and spinal cord.

## Data Availability

The datasets generated during and/or analyzed during the current study are available from the corresponding author on reasonable request.
