# Supplementary material for: The selective GSK3 inhibitor, SAR502250, displays neuroprotective activity and attenuates behavioral impairments in models of neuropsychiatric symptoms of Alzheimer’s disease in rodents
Source: Sci Rep. 2019 Dec 2;9:18045. doi: 10.1038/s41598-019-54557-5 (PMC6888874; doi:10.1038/s41598-019-54557-5)
Supplement: Supplementary file 1 — Western blot images of total tau and pS396-tau in P301L human tau transgenic mice cortex and spinal cord. [file 41598_2019_54557_MOESM1_ESM.docx]

**SUPPLEMENTARY MATERIAL**

**The selective GSK3 inhibitor, SAR502250, displays neuroprotective activity and attenuates behavioral impairments in models of neuropsychiatric symptoms of Alzheimer’s disease in rodents**

Guy Griebel, Jeanne Stemmelin, Mati Lopez-Grancha, Denis Boulay, Gerald Boquet, Franck Slowinski, Philippe Pichat, Sandra Beeské, Shinji Tanaka, Akiko Mori, Masatake Fujimura & Junichi Eguchi

**Figure S1**: Western blot images of total tau and pS396-tau in P301L human tau transgenic mice cortex and spinal cord.
